# Oat Protects against Diabetic Nephropathy in Rats via Attenuating Advanced Glycation End Products and Nuclear Factor Kappa B

**DOI:** 10.1155/2013/609745

**Published:** 2013-10-10

**Authors:** Abdulrahman L. Al-Malki

**Affiliations:** Department of Biochemistry, Faculty of Science, King Abdulaziz University, P.O. Box 80203, Jeddah 21589, Saudi Arabia

## Abstract

Oat, a rich source of soluble fiber, was considered to have a possible preventive effect on the progression of diabetic nephropathy. The present study aimed to assess this preventive activity in a rat model of diabetic nephropathy. Adult Wister rats were injected by streptozotocin (65 mg/kg). Animals were fed with normal diet or with a diet containing 20% oat (W/W) for 21 weeks. At the end of 21 weeks, all the kidney tissues were collected for various examinations. Our results suggested that oat could decrease the Scr and glucose level in blood of diabetic rats significantly (*P* < 0.05), and increase the creatinine clearance (*P* < 0.01). In histopathological examination, oat-fed rats showed a significant decrease in glomerulus segmented sclerosis and incidence of tubule vacuolar degeneration. By ELISA, we reported that oat feeding resulted in decreasing the levels of IL-6 and AGE in serum and kidney homogenate. In addition, the levels of oxidative stress markers were markedly improved as a result of oat feeding. Furthermore, using EMSA, we showed that oat attenuated the activation of NF-*κ*B. Using RT-PCR, we found that oat could downregulate the TGF-*β*1 and RAGE expression at mRNA levels. This study suggests that oat can suppress diabetic nephropathy in rats effectively and may slow down the renal fibrosis by the disruption of the detrimental AGE-RAGE-NF*κ*B axis.

## 1. Introduction

Diabetic nephropathy (DN) is the most common cause contributing to end-stage renal disease (ESRD) [1]. The chronic hyperglycemia destroys function and structure the kidney, leading to albuminuria which in turn further damages the renal tubular structure [2]. In diabetes, the kidney is a direct target to the enhanced glucose levels. Advanced glycation end products (AGE) are heterogeneous products formed by the nonenzymatic reactions between reducing sugars and free amino groups of proteins, lips, and nucleic acids [3]. 

AGE have been implicated as one of the major factors in the pathogenesis of numerous pathologies (aging, atherosclerosis, rheumatoid arthritis, and Alzheimer's disease) notably including diabetic complications [4–6]. AGE can interfere with protein function and promote formation of aggregates. In kidney, AGE can become trapped in glomerular basement membranes and covalently crosslink to collagen resulting in membrane thickening and distortion [7]. In addition to these direct effects, AGE can bind mesangial cell surface receptors stimulating formation of transforming growth factor *β*1 (TGF-*β*1) and connective tissue growth factor (CTGF), which in turn mediates mesangial expansion and glomerulosclerosis [8]. Furthermore, AGE interact with their receptor (RAGE) on the cell membrane resulting in activation of the nuclear transcription factor kappa B (NF-*κ*B). NF-*κ*B is found in the cytoplasm of the unactivated cells bound to its inhibitor I-*κ*B. Upon activation, NF-*κ*B migrates to the nucleus where it upregulates the transcription of several inflammatory genes like IL-6, TGF*β*, and others [9].

Many AGE inhibitors offer a promising therapeutic approach for diabetes-related complications [10, 11]. AGE-inhibitors include aminoguanidine (AG, Pimagedine) [12], pyridoxamine [13], aspirin [14], OPB9195 [15], and LR compounds [16]. Compounds that break AGE crosslink include phenacylthiazolium bromide (PTB) [17] and ALT-711 (Alagebrium) [18]. Some of the agents targeting AGE have been approved for clinical trials [19].

 Cumulative studies demonstrated that dietary fiber can significantly reduce the risk of cardiovascular disease and type 2 diabetes mellitus [20]. This is due in part to the ability of fiber to reduce postprandial glycaemia and improve long-term glycemic control [21, 22]. It was postulated that the rheological properties of soluble dietary fibers are highly related to their effects on control of the glucose concentration [23]. For instance, the ability of oat-derived *β*-glucan to reduce postprandial glycaemia has been strongly correlated with its viscosity [24], demonstrating an inverse linear relationship between the logarithm of viscosity measures and peak postprandial plasma glucose and insulin responses after consuming various doses of purified oat *β*-glucan with a 50 g oral glucose load. Despite these findings, the levels of viscosity required to achieve specific glucose-lowering effects are poorly understood. Still, the majority of trials investigating dietary fiber have not accounted for the principles of polysaccharide solubility and viscosity as the main determinants of its physiological outcome. While a small number of studies have shown the effect of oat on diabetes [25, 26], none examined its effect on the development and progression of diabetic nephropathy.

The aim of this study is to evaluate the effect of oat on the hyperglycemia-induced AGE formation and NF-*κ*B activation and if this can attenuate the development of diabetic nephropathy. Because oat is natural dietary supplement and widely used, the results of this study may provide an alternative for enhancing nutrition and diabetic control during diabetic nephropathy.

## 2. Materials and Methods

### 2.1. Animals

Adult male Wistar rats (Rattus Rattus) weighing about 190 ± 30 g were used in the present study. All animals were housed in cages and received normal rat diet and tap water *ad libitum* in a constant environment (room temperature 24 ± 3°C, room humidity 55 ± 5%) with a 12 h light, 12 h dark cycle. The animals were kept under observation for two weeks prior to the start of the experiments. 

### 2.2. Induction of Diabetes Model and Study Design

Forty-five Wister rats were used in this experiment. Ten rats were used as normal control group (group 1, *n* = 10), which received a single *ip* injection of 0.1 mol/L citrate buffer. A group of 35 rats were intravenously injected with STZ (65 mg/kg body weight) [27] in a 0.1 mol/L citrate buffer (pH 4.5). Only rats with blood glucose higher than 300 mg/dL after 7 days were considered as being diabetic in the fasting state. Glucose measurement was done by using *OneTouch Select* Analyzer (LifeScan, Inc., UK). Rats with blood glucose lower than 250 mg/dL were excluded from the study (6 rats). All studies were carried out two days after induction of diabetes. Twenty-nine diabetic rats were randomly divided into two groups: diabetic untreated rats (*n* = 15 rats) and diabetic rats that received 20% oat in the diet (W/W) (*n* = 14) [28]. Rats were fed with normal rat meal and oat containing diet 20 g for each rat/day for 21 weeks [29]. Body weight blood glucose and HbA1C levels were measured regularly and at the end of the experiment duration. At the end of the experiment (21 weeks after induction of diabetes), animals were sacrificed. Kidneys were dissected and rinsed with ice cold normal saline and then weighed. An index of renal hypertrophy was estimated by comparing the wet weight of the left kidney to the body weight. 

### 2.3. Kidney Homogenate Preparation

One kidney of each group was washed by cold normal saline solution, then it was homogenized in a homogenization buffer (0.05 M Tris-HCl pH 7.9, 25% glycerol, 0.1 mM EDTA, and 0.32 M (NH_4_)_2_SO_4_) containing a protease inhibitor tablet (Roche, Germany). The resulting solution was sonicated in an ice bath for 10 seconds followed by centrifugation at 13000 rpm, 4°C for 5 minutes. The supernatant was aliquoted and stored at −80°C and assayed for protein concentration using BCA kit (Pierce, Rockford, USA) using bovine serum albumin diluted in the lysis buffer as standard. The homogenate was used for the determination of antioxidant biomarkers, concentration of AGE, and level of IL-6 and TGF*β*. The other kidney from each group was used for histopathological, isolation of renal DNA, extraction of the nuclear proteins [30].

### 2.4. Determination of Serum Biomarkers

Blood sample of rats was centrifuged at 8000 rpm for 10 minutes at 4°C, and serum was removed and aliquoted for the respective analytical determinations. The diagnostic kits for determinations of creatinine (Cr) blood urea nitrogen (BUN), sodium, and potassium were purchased from BioSystem (Barcelona, Spain). All analyses were performed according to the instructions of the manufacturer.

### 2.5. Analysis of Urine Parameters

Before induction of diabetes and the day before the end of the experiment, urine samples were collected by placing the rats in individual metabolic cages for 24 h. The urine albumin concentration was determined using an ELISA kit (Nephrat II, Exocell, Philadelphia, PA, USA) and the concentration of Cr in pooled urine samples was determined by the commercial assay kit. All analyses were performed in accordance with the manuals provided by the manufacturers. The 24 h urinary albumin excretion rate (UAER) was calculated as UAER (*μ*g 24 h − 1) = urinary albumin (**μ**g mL^−1^) × 24 h urine volume (mL). Cr clearance (Ccr) was calculated using the following equation: Ccr (mL min^−1^ kg^−1^) = [urinary Cr (mg dL^−1^) × urinary volume (mL)/serum Cr (mg dL^−1^)] × [1000/body weight (g)] × [1/1440 (min)] [31]. 

### 2.6. Determination of the Antioxidant Biomarkers

The activities of total SOD, catalase, and GSH-Px as well as the concentrations of MDA and GSH in the kidney homogenate were determined using commercially available kits from BioVision Research Products (Linda Vista Avenue, USA) according to the instructions of the manufacturer [32–36]. 

### 2.7. Determination of IL-6 and TGF*β*

The levels of IL-6 and TGF*β* in the serum and in the kidney homogenate were assayed by using the commercially available ELISA kits from R&D (Mannheim, Germany) according to the instructions of the manufacturer. 

### 2.8. Measurement of Urinary and Renal 8-Hydroxy-2′-Deoxyguanosine

Urinary 8-hydroxy-2′-deoxyguanosine (8-OHdG) levels were determined using an ELISA kit from Genox Corporation (Baltimore, MD, USA) according to the method of Vuksan et al. [21] and corrected by using individual urine creatinine concentrations. Extraction of renal DNA was performed using a DNA extraction kit (Promega, Germany) according to the manufacturer's protocol. The genomic DNA samples from kidney tissue were also used for the determination of 8-OHdG using the competitive ELISA kit [37].

### 2.9. Determination of AGE in the Kidney Homogenate

The renal AGE level was determined according to previous method [38]. The concentration of AGE was determined by a competitive ELISA assay. The ELISA kit for AGE determination was obtained from Roche Diagnostics (Mannheim, Germany). As a standard, the monomeric epitope N-carboxymethyl-aminocaproate was used.

### 2.10. Electrophoretic Mobility Shift Assay (EMSA)

Part of the kidney of each group was dissolved in TOTEX buffer (100 mM HEPES-KOH, pH 7.9, 20% glycerol, 0.35 M sodium, chloride, 1% NP 40, 1 mM magnesium chloride, EGTA 0.5 mM, EDTA 0.5 mM, 10 *μ*g/mL leupeptin, 0.2 mM PMSF, and 0.5 mM DTT) for 30 seconds. The lysate was incubated in ice for 0.5 h, vortexed, and centrifuged at 10000 g for five minutes. The supernatant which contained the total liver extract was transferred to a fresh tube and kept at −80°C for EMSA [25]. The extract was used for determining of the binding activity using the NF-*κ*Bp65 consensus sequence: 5′-AG TT GA GG GG AC TT TC CC AG GC-3′. The binding specificity of the mixture was ascertained by competition with a 160-fold molar excess of the unlabeled consensus oligo nucleotides as previously described [39, 40]. Incubation was carried out for 30 min at room temperature in a total volume of 15 mL of buffer that contains 12 mM HEPES/NaOH, pH 7.9, 12% glycerol, 60 mM potassium chloride, EDTA 1 mM, dithiothreitol 1 mM, 1.0 mg of poly (dI-dC), and 10,000 cpm of the labeled probe. The DNA/protein complexes were resolved on nondenaturing 5% polyacrylamide gel electrophoresis, performed with 0.5x Tris/borate/EDTA buffer (4.5 mM boric acid, 0.1 mM EDTA, pH 8.0, and 4.5 mM Tris) [41]. The increase in the expression of NF*κ*Bp65 gene was determined by a Phosphor Imager using background substract.

### 2.11. Reverse Transcription Polymerase Chain Reaction

Total RNA of rats in the three groups was isolated from kidney cortex by using TRIzol reagent (Invitrogen, Darmstadt, Germany) according to the manufacturer's protocol. Gene expressions were determined using real-time quantitative reverse transcription polymerase chain reaction using light cycler and cyber green kit from Roche (Mannheim, Germany). *β*-actin was used as a reference gene. The transcripts were amplified in a single reaction containing 1 mg cDNA and 0.5 mM each of the sense and antisense primers. PCR was performed at 94°C 1 cycle for five minutes, followed by 33 cycles at 94°C 1 cycle 1 minute, 62°C 1 cycle 1 minute, 72°C 1 cycle 1 minute, and a final extension step at 72°C 1 cycle 20 minutes. An 08% agarose gel electrophoresis in presence of ethidium bromide as a stain was used for separation of the PCR products. A Digital Imaging System (Gel Logic 2200 Pro, Kodak, USA) was used for scanning and analyzing the obtained bands. The band intensities were expressed relative to the intensity of the bands of the control group which was set as 100%. The sequences of the used primers were as follows:

 
*TGF*β*1* sense 5′-TGGAGCAACATG TGG AAC TC-3′, antisense 5′-GTC AGC AGC CGG TTA CCA-3′;
 
*RAGE* sense 5′-CAC  AGC  CCG  GAT  TG-3′, antisense 5′-GCT  GTA  GCT  GGT  GGT  CAG  AAC  A-3′;
 
**β*-actin* sense 5′-GTGCTATGTTGCTCTAGACTTCG-3′, antisense 5′-ATGCCACAGGATTCCATACC-3′.

### 2.12. Histopathological Examination

Part of the kidneys was fixed in a 10% neutral formaldehyde solution and embedded in paraffin. Sections were cut at 4 mm with a microtome and deparaffined with xylene. They were stained with hematoxylin and eosin (H&E) staining. Stained kidney sections were observed under a light microscope at magnifications of 200x and 400x [42].

### 2.13. Statistical Analysis

All group values are expressed as the mean ± SD. Data were evaluated using IBM SPSS 20 for windows. An analysis of variance test was performed initially to test for differences in the treatment, and a Tukey post hoc test and Student's *t*-test were performed to examine whether there were any significant differences between the three groups. 

## 3. Results

### 3.1. Effects of Oat Feeding on the Blood Glucose and Kidney/Body Weight Ratio

As indicated in Table 1, STZ injection resulted in a nearly 6-fold increase of the fasting blood sugar (FBS) in the rats of group 2. In addition, STZ injection caused a nearly 3-fold increase in the levels of HbA1C. Oat feeding markedly reduced the elevated levels of FBS and HbA1C. The final body weight of the diabetic untreated animals was markedly decreased compared with the normal control rats indicating that these rats suffer from growth retardation as a result of STZ injection. At the end of the experimental period, the kidney/final body weight ratios of the untreated diabetic animals were significantly higher than those of the control rats (*P* < 0.01). As a result of oat feeding, the diabetic animals in the third group showed a significant reduction of this elevated ratio compared to the diabetic animals of group 2 (*P* < 0.05).

### 3.2. Effects of Oat Feeding on the Renal Function

Data in Table 1 showed that the 24-hour Upro, creatinine, Ccr, potassium, and sodium levels were markedly higher in the diabetic untreated group when compared with the normal control group (*P* < 0.05). Oat feeding obviously reduced these elevated renal biomarkers in the animals of group 3 compared with rats of group 2 (*P* < 0.05).

### 3.3. Effects of Oat on Activities of Oxidant/Antioxidant Enzymes

The activity of SOD, catalase, GSH-px, and GST as well as the concentration of GSH was markedly reduced, whereas the concentration of MDA was markedly increased in the kidney homogenate of the diabetic untreated rats (group 2) compared to the control group (group 1) (*P* < 0.05), suggesting that these rats suffered from oxidative stress (Table 2). As a result of oat feeding, these altered parameters were significantly improved in the animals of group 3 (*P* < 0.05). The obtained data showed that oat ameliorated oxidative stress in the diabetic rats.

### 3.4. Effects of Oat on the Urinary and Renal 8-OHdG

**Figure 1:**
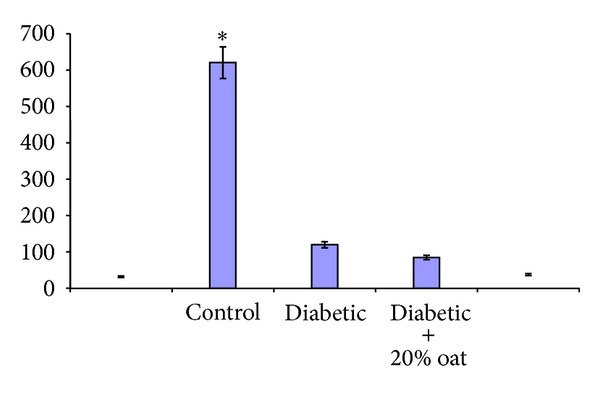
The levels of 8-hydroxy-2′-deoxyguanosine in the urine (a) and renal cortex (b) of rats. The levels of the urinary and renal cortex 8-OHdG in diabetic rats were markedly reduced as a result of oat feeding. Data are expressed as the means ± SD. *P < 0.05 versus normal control group, **P < 0.05 versus diabetes untreated group.

The total amounts of urinary 8-OHdG were significantly greater in diabetic rats (group 2) than in the control rats after 21 weeks after the onset of diabetes (*P* < 0.05). Feeding of oat resulted in the suppression of the increase in urinary excretion of 8-OHdG in the diabetic rats (*P* < 0.05). In parallel with the urine results, the levels of 8-OHdG in the DNA were markedly increased in the kidney cortex of the diabetic rats. As shown in Figure 1, all the elevated levels of 8-OHdG were normalized by oat feeding (*P* < 0.05). 

### 3.5. Effect of Oat on the Levels of AGE

As a result of diabetes, the kidneys of diabetic rats are subjected to elevated levels of glucose. The renal levels of AGE and CML were significantly elevated in the STZ-diabetic rats. These elevated levels were effectively lowered by oat feeding for 21 weeks (Table 2). 

### 3.6. Effect of Oat on the Activation of NF-*κ*B

**Figure 2:**
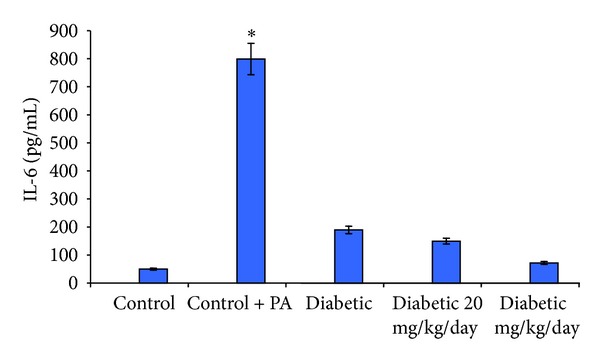
Binding activity of NF-*κ*B. Binding activity of NF-*κ*B to its consensus sequence was assayed by EMSA of total nuclear protein extracts. Quantification of activated NF-*κ*B was performed by densitometric analysis of relative EMSA band intensities. Results are the means ± SE of four individual replicates, value from an unpaired Student's *t*-test (*P < 0.05, compared with normal control group and **P < 0.001 comparing with diabetic untreated group).

As a result of diabetes, oxidative stress, and high levels of AGE, NF-*κ*B-p65 was markedly activated in the diabatic untreated animals compared with the normal control. Feeding of the diabetic rats with oat in group 3 resulted in a significant reduction of the NF-*κ*B-p65 as indicated in Figures 2(a) and 2(b). 

### 3.7. Effects of Oat Feeding on RAGE and TGF-*β* in Rats Kidneys

**Figure 3:**
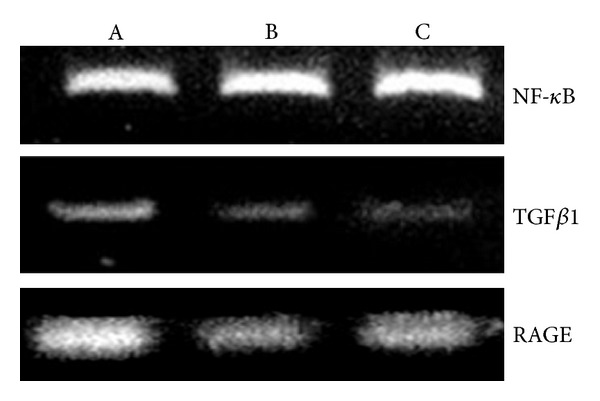
Expression of NF-*κ*B, TGF*β*1, and RAGE in the kidney of control, diabetic, and diabetic + oat rats. RT-PCR analysis by using total RNA extracted from the kidney cortex is shown. Data represent the means with SEs of three independent experiments from three different rats. Gene expression is illustrated as a ratio of the integrated density values for the genes in question and the integrated density of the actin values to yield a semiquantitative assessment. A: control, B: diabetic, and C: diabetic + 20% oat.

The expression of RAGE and TGF-*β*1 was significantly higher in the diabetic untreated group compared with the normal control animals. Oat feeding of the animals in group 3 markedly attenuated the expression of both genes compared with the diabetic untreated rats (group 2) as illustrated in Figure 3.

### 3.8. Effect of Oat Feeding on Serum and Renal IL-6

As a result of diabetes, inflammation increases and the release of cytokines like IL-6 increases. Data in Table 1 showed that the serum level of IL-6 was markedly increased as a result of diabetes in group 2 compared with the normal control group (*P* < 0.05). In parallel, the level of IL-6 in the kidney homogenate of rat in group 2 was significantly increased (*P* = 0.012). Rats of group 3 which fed oat containing diets showed a significantly lower IL-6 levels in both serum and kidney homogenate (Tables 1 and 2) (*P* < 0.05). 

### 3.9. Histopathological Findings

**Figure 4:**
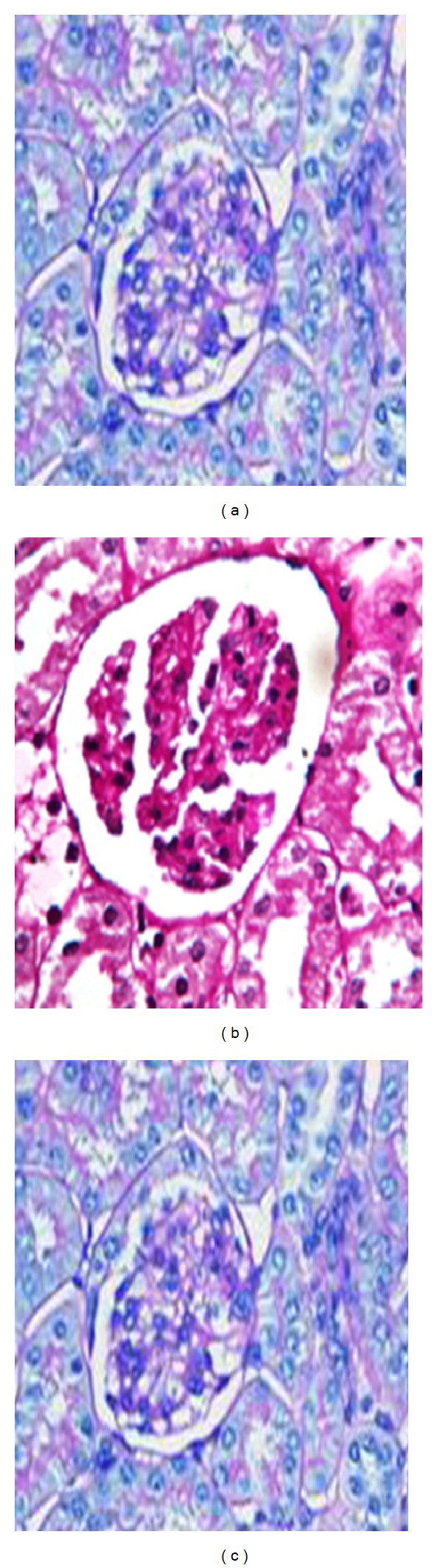
Mesangial expansion and ultra structural changes in diabetic apoE−/− mice after 12 weeks after induction of diabetes. (a) Nondiabetic mice without PA. (b) Diabetic mice without PA. (c) Diabetic mice with PA. PAS staining, original magnification x200.

After feeding with oat for 21 weeks, all the kidneys were collected for histological examination. In diabetes control group, the renal lesions mainly existed in glomerulus and renal tubules. As compared with the diabetes control rats, oat-fed rats showed significantly less severe sclerosis in glomerulus segments. Nevertheless, oat reduced the incidence of tubule vacuolar degeneration and severity level, suggesting some preventive effect on renal tubule lesions. Representative photomicrographs of glomerular changes were shown in Figure 4. The major differences in diabetes controls and oat-fed animals were segmental and diffuse mesangial expansion of the glomeruli. Hypocellular, sclerotic lesions compatible with diabetic glomerulosclerosis were observed in glomeruli of the diabetic animals under a light microscope.

## 4. Discussion

In the present study, 21 weeks diabetes by STZ produced a diabetic nephropathy which was manifested by increased creatinine, creatinine clearance, serum BUN, and 24 h urinary albumin. Feeding with Oat significantly reversed the alterations of renal function and confers a hypoglycemic effect which contributes at least in part to a reversal of diabetic nephropathy. An increment in the urine albumin excretion in 24 h was significantly aggravated in diabetic nephropathy that is an important sign to indicate progressive damage to glomerular and tubular cells in diabetic kidney. Oat feeding slows down the 24 hour urinary albumin excretion, in association with a significant decline in BUN and creatinine and creatinine clearance in serum, respectively.

Hyperglycemia causes an increased production of free radical which plays a major role in the disruption of the cellular functions of the kidney that correlates to a decline in the endogenous ROS scavengers such as GSH-px, SOD, GST and catalase (Kalia et al., 2004; Cameron et al., 2005; Jandeleit-Dahm et al., 2005). Oxidative stress as indicated by the high levels of 8-OHdG and the overproduction of the superoxide anions is implicated in the pathophysiology of diabetic nephropathy (Vural et al., 2002). Oat feeding confers the benefit to suppress MDA and 8-OHdG production. In addition, the activity of GST, GSH-px catalase, and SOD was elevated which results in the reduction of progression of the renal lesions. 

The overproduction of ROS in diabetes results directly in the enhanced formation of AGE [3]. One important receptor for mediating AGE effects is RAGE [24]. RAGE is expressed in the diabetic kidney, both in tubular and in glomerular cells [25]. Binding of AGE such as CML to RAGE augments and propagates oxidative stress in the kidney tissue [25, 26]. RAGE signals via the transcription factor NF*κ*B to activate target genes which have deleterious potential for the diabetic kidney [23, 27, 28]. Feeding with oat reduced RAGE expression, NF*κ*B p65 antigen, and NF*κ*B binding activity. The pivotal role of RAGE in the mediation of ROS/AGE-induced diabetic microangiopathy is supported by experiments using transgenic and knockout mice. RAGE-null mice are protected from diabetes-induced kidney damage (H.-P. Hammes, A. Bierhaus et al., unpublished observations). Thus, our data suggest that part of the beneficial effect of oat includes the disruption of the detrimental AGE-RAGE-NF*κ*B axis.

Activation of NF*κ*B results in its migration into the nucleus. In the nucleus, it upregulates the transcription of its controlled genes like IL-6, TGF*β*, and so forth. In the diabetic group, the levels of IL-6 in both serum and kidney homogenate as well as the levels of TGF*β* were markedly increased. The results are in line with previous data in parallel with the NF*κ*B results in Figure 2. Feeding with oat reduced AGE and NF*κ*B activation and this resulted in the attenuation of the levels of both cytokine. 

Diabetic nephropathy is caused by many factors and cannot be sufficiently controlled with a strict control of hyperglycemia. The early stage diabetic nephropathy is induced by ambient hyperglycemia, but secondary effects are not dependent on persistent hyperglycemia (Vestra and Fioretto, 2003) and it is therefore not enough to merely control serum glucose levels in order to retard the development of diabetic nephropathy. The level of oxidative stress, AGE and NF-*κ*B, together with the elevated levels of cytokine llike IL-6 and TGF*β*1, play crucial roles in the functional and pathological damage to the kidney. Feeding with oat slows down the progression of the disease progression by normalizing these abnormalities. 

In summary, we provide evidence that ROS scavenging is an effective approach for the prevention of diabetic nephropathy. Oat is a paradigm food supplement with a broad spectrum of beneficial effects, based on its ability to reduce the sequelae of hyperglycaemia-induced ROS overproduction. Since oat also has beneficial effects on other target tissues of diabetic angiopathy and shows beneficial effects on mediators of large vessel damage, this concept appears attractive for the prevention or delay of diabetic angiopathy.

## Figures and Tables

**Table 1:** Biochemical, physiological, and renal functional parameters of the rats.

	Normal control, *n* = 10	Diabetic untreated, *n* = 9	Diabetic plus 20% oat, *n* = 13
Initial body weight, g	201.3 ± 5.6	202.4 ± 4	199.7 ± 9
Final body weight, g	271.5 ± 8.1	159.5 ± 8.2^a^	224 ± 12.3^a,b^
Glucose, mg/dl	99.98 ± 5.48	530.55 ± 20^a^	237.5 ± 28^a,b^
HbA1c, %	5.11 ± 0.15	13.42 ± 0.34^a^	8.95 ± 0.9^a,b^
Kidney/body weight, g/g, ×10^−3^	6.45 ± 0.65	11.15 ± 1.76^a^	8.12 ± 0.9^a,b^
BUN, mmol/L	7.23 ± 0.98	14.95 ± 2.91^a^	8.62 ± 2.1^a,b^
Serum creatinine, mmol/L	50.45 ± 8.2	69.52 ± 7.97^a^	53.15 ± 4.5^b^
Ccr mLmin^−1^ kg^−1^	3.51 ± 0.3	6.78 ± 0.91^a^	3.43 ± 0.56^b^
U prot, mg/24 h	8.53 ± 0.93	22.56 ± 3.1^a^	14.95 ± 3.12^a,b^
Serum sodium, mmol/L	144.4 ± 3.5	176.1 ± 10.5^a^	150.1 ± 1.2^b^
Serum potassium, mmol/L	4.54 ± 0.83	7.76 ± 0.76^a^	5.34 ± 0.42^b^
Serum IL-6, g/mL	82.2 ± 0.54	695.2 ± 16.46^a^	211.2 ± 13.34^a,b^

Data are expressed as the means ± SD. ^a^ *P* < 0.05 versus normal control group, ^b^ *P* < 0.05 versus diabetic untreated group, one way ANOVA.

**Table 2:** Oxidant/antioxidant parameters as well as concentration of AGE and IL-6 in the rat kidney homogenate.

	Normal control, *n* = 10	Diabetic untreated, *n* = 9	Diabetic plus 20% oat, *n* = 13
MDA, nmol/mg protein	4.04 ± 0.1	9.76 ± 1.3^a^	5.2 ± 0.65^b^
GST, nmol/mg protein	19.76 ± 2.05	11.1 ± 1.23^a^	15.97 ± 2.1^a,b^
GSH-Px, U/mg protein	0.99 ± 0.23	0.29 ± 0.21^a^	0.75 ± 0.21^b^
Catalase, U/mg protein	52.16 ± 3.85	31.76 ± 2.5^a^	42.74 ± 3.95^a,b^
SOD, U/mg protein	25.17 ± 4.2	9.54 ± 1.91^a^	17.65 ± 2.34^b^
GSH, nmol/mg protein	27.76 ± 3.27	14.11 ± 1.95^a^	19.54 ± 3.56^b^
AGE, ng/mg protein	3.91 ± 0.12	9.75 ± 0.54^a^	5.11 ± 0.25^b^
IL-6, ng/mg protein	255 ± 22	1145 ± 69^a^	564 ± 48^a,b^

Data are expressed as the means ± SD. ^a^ *P* < 0.05 versus normal control group, ^b^ *P* < 0.05 versus diabetic untreated group, one way ANOVA.
